# Circulating Irisin Is Reduced in Male Patients with Type 1 and Type 2 Myotonic Dystrophies

**DOI:** 10.3389/fendo.2017.00320

**Published:** 2017-11-14

**Authors:** Elena Dozio, Elena Passeri, Rosanna Cardani, Stefano Benedini, Carmen Aresta, Rea Valaperta, Massimiliano Corsi Romanelli, Giovanni Meola, Valeria Sansone, Sabrina Corbetta

**Affiliations:** ^1^Department of Biomedical Sciences for Health, University of Milan, Milan, Italy; ^2^Endocrinology and Diabetology Service, IRCCS Istituto Ortopedico Galeazzi, Milan, Italy; ^3^Laboratory of Muscle Histopathology and Molecular Biology, IRCCS Policlinico San Donato, Milan, Italy; ^4^Research Laboratories, IRCCS Policlinico San Donato, Milan, Italy; ^5^Laboratory of Medicine Unit SMEL-1, IRCCS Policlinico San Donato, Milan, Italy; ^6^Neurology Unit, IRCCS Policlinico San Donato, Milan, Italy; ^7^Centro Clinico Nemo, Neurorehabilitation Unit, Milan, Italy

**Keywords:** myotonic distrophies, myotubes, irisin, fat mass, insulin resistance, bone density

## Abstract

**Context:**

Myotonic dystrophies (DM) are dominantly inherited muscle disorders characterized by myotonia, muscle weakness, and wasting. The reasons for sarcopenia in DMs are uncleared and multiple factors are involved. Irisin, a positive hormone regulator of muscle growth and bone, may play a role.

**Objectives:**

To investigate (1) circulating irisin in a series of DM1 and DM2 male patients compared with healthy controls and (2) the relationships between irisin and anthropometric, metabolic and hormonal parameters.

**Design and study participants:**

This is a cross-sectional study. Fasting blood samples for glucometabolic, gonadic, bone markers, and irisin were collected from 28 ambulatory DM1, 10 DM2, and 23 age-matched healthy male subjects. Body composition and bone mineralization [bone mineral density (BMD)] were measured by DEXA. Echocardiographic assessment and visceral adiposity, namely, liver and epicardial fat, were investigated by ultrasound. Irisin released from cultured myotubes derived from 3 DM1, 3 DM2, and 3 healthy donors was assayed.

**Results:**

Plasma irisin levels were definitely lower in both DM1 and DM2 patients than in controls with no difference between DM1 and DM2. Irisin released from DM1 and DM2 myotubes was similar to that released from myotubes of the non-DM donors, though diabetic DM2 myotubes released more irisin than DM1 myotubes. There was no correlation between irisin and muscle strength or lean mass in both DM1 and DM2 patients. In DM1 patients, plasma irisin levels correlated negatively with oxygen consumption and positively with insulin resistance, while in DM2 patients plasma irisin levels positively correlated with fat mass at arms and legs levels. No correlation with visceral fat, left ventricular mass, and gonadal hormones could be detected. In both DM1 and DM2 patients, legs BMD parameters positively correlated with plasma irisin levels.

**Conclusion:**

Plasma irisin is reduced in both DM1 and DM2 male patients likely reflecting muscle mass reduction. Moreover, insulin resistance may contribute to modulation of plasma irisin in DM1 patients. The irisin-mediated cross talk muscle–adipose tissue–bone may be active also in the male myotonic dystrophies’ model.

## Introduction

Skeletal muscle is emerging as an endocrine organ. A number of biological active molecules are expressed and released from muscle cells known as myokines (1). Among them, irisin, the cleaved fragment of the transmembrane protein type-III domain containing protein 5 (FNDC5), was shown to induce adipocyte browning. Irisin acts on subcutaneous adipose tissue increasing thermogenesis and energy expenditure, therefore granting protection against obesity and insulin resistance (2). Moreover, irisin stimulates myogenesis (3). The circulating 22 kDa form of irisin has been quantitated in human plasma by mass spectrometry (4, 5). Plasma irisin levels are increased in mice and humans after short-term exercise (6), and it is emerging as a sensitive marker for muscle weakness and atrophy (7).

Myotonic dystrophies (DM) are multisystemic disorders affecting skeletal muscles, with myotonia, muscle weakness and atrophy (8). Myotonic dystrophy type 1 (DM1) is the most common adult form of muscular dystrophy, while myotonic dystrophy type 2 (DM2) is rare. DM1 is due to a CTG repeat expansion in the *DMPK* gene on chromosome 19q13, while an expansion of CCTG repeat in the *CNBP* gene on chromosome 3q21 causes DM2 (9). Multiple organs are involved including pancreas, liver, gonads, thyroid, and bone. Insulin resistance, liver steatosis, hypogonadism, goiter, and vitamin D deficiency are common features. Sarcopenia is multifactorial, though it is likely that the endocrine abnormalities play a role (10). At the skeletal muscle level, still there is no mechanistic explanation for the observed muscle weakness and atrophy in DM patients (11–13).

Here, we tested the hypothesis that DM-related myopathic changes of skeletal muscle cells might impair their endocrine function, therefore contributing to the non-muscle phenotype of both DM conditions, namely, insulin resistance, lipid profile alterations, visceral fat distribution, and bone mineral impairment. Therefore, we investigated the circulating and myotubes-released irisin in DM patients compared with non-DM donors.

## Materials and Methods

### Study Population

Twenty-eight male patients (44.7 ± 11.6 years; mean ± SD) affected with adult-onset DM1, 10 male patients affected with DM2 (56.7 ± 9.3 years) and 23 age-matched, physically active, male healthy control subjects (49.5 ± 8.3 years) were consecutively enrolled. We focused the study in male patients to avoid gender differences in the gonadal hormonal status. Clinical and bioptic diagnosis of DM1 was genetically confirmed by Southern blot-based kit (14). Clinical diagnosis of DM2 was confirmed by both fluorescence *in situ* hybridization on muscle frozen sections using a (CAGG) 5 probe and by Southern blot-based kit (15).

All the DM patients were ambulant. Patients aged <18 and >70 years, with heart, kidney, or liver failure, previous or current corticosteroid treatment, hormone replacement, anti-epileptic therapy were excluded. All the participants gave their written informed consent and the local ethical committee approved the study protocol. The study complied with the Declaration of Helsinki.

### Clinical, Biochemical, and Hormonal Assessment

Anthropometric measurements were investigated in all patients and healthy controls, including height, weight, and body mass index. Abdominal fat was evaluated as waist circumference, liver fat, and epicardic fat were evaluated by ultrasound imaging, as previously described (16). Calculation of resting energy expenditure was performed by indirect calorimetry (VMAX Encore, VIASYS Healthcare, Inc., Yorba Linda, CA, USA). All DM patients were studied by routine ultrasound cardiac imaging for the evaluation of cardiac mass. Muscle strength was determined according to the modified 5-point MRC scale (Medical Research Council) in the upper and lower limbs for a total of 150 maximum score (Medical Research). Stage of the disease for DM1 patients was defined using Muscular Impairment Rating Scale (17). Mobility was assessed according to Rivermead Mobility Index (RMI) (18). A subgroup of 15 DM1 and 10 DM2 patients was further investigated by dual-energy X ray absorptiometry (DEXA) total body scanner for regional body composition and for the measurement of segmental bone mineral density (BMD) using a Hologic densitometer. Regarding the lower and upper limbs, we considered a mean value of the BMDs measured at left and right limbs. Participants were scanned in light clothing, while lying flat on their backs with arms at their sides.

Venous blood samples were collected after an overnight fasting in all patients for determination of glucose, total and HDL cholesterol, triglycerides, albumin, HbA1c levels according to routinely used laboratory kits. To assess calcium and bone metabolism, calcium, phosphate, creatinine albumin and alkaline phosphatase were measured according to routinely used laboratory kits. Serum PTH levels were determined by ElectroChemiLuminescence on an Elecsys 2010 (Roche Diagnostics, Mannheim, Germany) and serum 25OHvitamin D (25OHD) was measured by a chemiluminescent assay (LIAISON^®^ test, DiaSorin Inc., Stillwater, MN, USA). Gonadic function was assessed by measurement of serum total testosterone (T), SHBG, 17βestradiol, LH, and FSH levels by ECLIA assays (Roche Diagnostic, Milan, Italy). Free testosterone (free T) was calculated from T, SHBG and albumin according to the method of Vermeulen et al. (19). Serum anti-Müllerian hormone (AMH) and inhibin B concentrations were measured by a II generation ELISA kit (Beckman Coulter, Brea, CA, USA).

Plasma irisin levels were measured by an irisin/FNDC5 (extracellular domain molecule: epitope 16-127) assay kit (Phoenix Pharmaceuticals, CA, USA; sensitivity 1.3 ng/ml, range 0.1–1,000 ng/ml and linear range between 1.29 and 27.5 ng/ml) (20, 21). Antibody used in this kit recognizes recombinant full length irisin, irisin (42–112) (100%) and recombinant FNDC5, isoform 4 (9%), but not the irisin precursor C-terminal 48-mer FNDC5 (165–212) and irisin (42–95). Irisin concentrations in conditioned media were determined using the same kit.

### Primary Human Skeletal Muscle Cell Cultures

Human satellite cells were isolated from biceps brachii muscle biopsies from 3 DM1, 3 DM2 patients and from three subject with no sign of neuromuscular disease used as controls, as previously described (22). Myoblasts were grown in HAM’s F10 medium (Sigma-Aldrich) supplemented with 15% FBS (Euroclone), 0.5 mg/mL albumin from bovine serum (BSA, Sigma-Aldrich), 0.5 mg/mL fetuin (Sigma-Aldrich), 0.39 µg/mL dexamethasone, 10 ng/mL epidermal growth factor, 0.05 mg/mL insulin, 3 mg/mL glucose, 100 U/mL penicillin, and 100 µg/mL streptomycin (proliferative medium). For this study, cells from DM and control patients were plated at a density of 60,000 cells per 35 mm dishes. When myoblasts reached 80% of confluence, proliferative medium was replaced by differentiative medium (DMEM/High Glucose supplemented with 7% FBS, in presence of 100 U/mL penicillin, 100 µg/mL streptomycin, and 0.01 mg/mL insulin) to allow myoblasts fusion. Myotubes and conditioned media were harvested after 5 days of differentiation (T5) and irisin concentration was determined after 6 h of incubation in serum and fetuin-free conditioned media.

### Immunofluorescence, Myogenic Purity, and Differentiative Capability

Immunofluorescence has been performed on proliferating myoblasts and on T5 myotubes Muscle cell cultures were fixed in 4% paraformaldehyde for 15 min at 4°C. After fixation, cells were washed several times in PBS and then permeabilized in 0.4% Triton X 100 in PBS for 5 min. After washing in PBS, non-specific binding sites were blocked with NGS (Dako-Cytomation) at a dilution 1:20 in PBS + 2% BSA for 20 min at room temperature. Myoblast and T5 myotubes were then incubated, respectively, with a primary antibody mouse monoclonal anti-desmin (CD33, Dako, 1:100 in PBS + 2%BSA) and a mouse monoclonal anti MHC-fast (Sigma- Aldrich, 1:600 in PBS + 2%BSA) for 1 h at room temperature. After washing in PBS 3× 5 min, cells were incubated for 1 h at room temperature with secondary antibodies (goat anti mouse Alexa 488-labeled; Molecular Probes, Eugene, OR, USA; 1:400 in PBS + 2%BSA). After washing in PBS 3× 5 min, nuclei were stained with 165 nM 4,6-diamidino-2-phenylindole. Cells were finally mounted with Mowiol and examined using a fluorescence microscope. Myogenic purity was evaluated on desmin immunostained myoblasts. The percentage of desmin positive myoblasts was calculated as the number of positive cells vs the total number of cells observed. Differentiative capability was evaluated as fusion index on T5 myotubes MHC-fast myosin immunostained. Fusion index was determined as number of nuclei in multinucleated myotubes expressed as a percentage of the total number of nuclei. At least 100 nuclei were counted in at least 10 different optical fields randomly chosen.

### Statistical Analysis

Continuous variable are presented as mean ± SD; normal distribution of continuous variables was tested: plasma irisin, insulin and HOMA-IR levels failed to pass normality test, therefore irisin values were normalized by log2 transformation for further statistical analysis. Simple correlation analyses were performed using Pearson correlation. Categorical data are presented as percentages. Fisher exact test or χ^2^ test were also used to compare the categorical variables among the different groups. Groups were compared using T test for normal variables or Mann–Whitney *U*-test for non-parametric variables. A *P* value less than 0.05 was considered significant. Statistical analysis was performed using Prism 6.0.

## Results

### Circulating Irisin in DM1 and DM2 Male Patients

**Figure 1:**
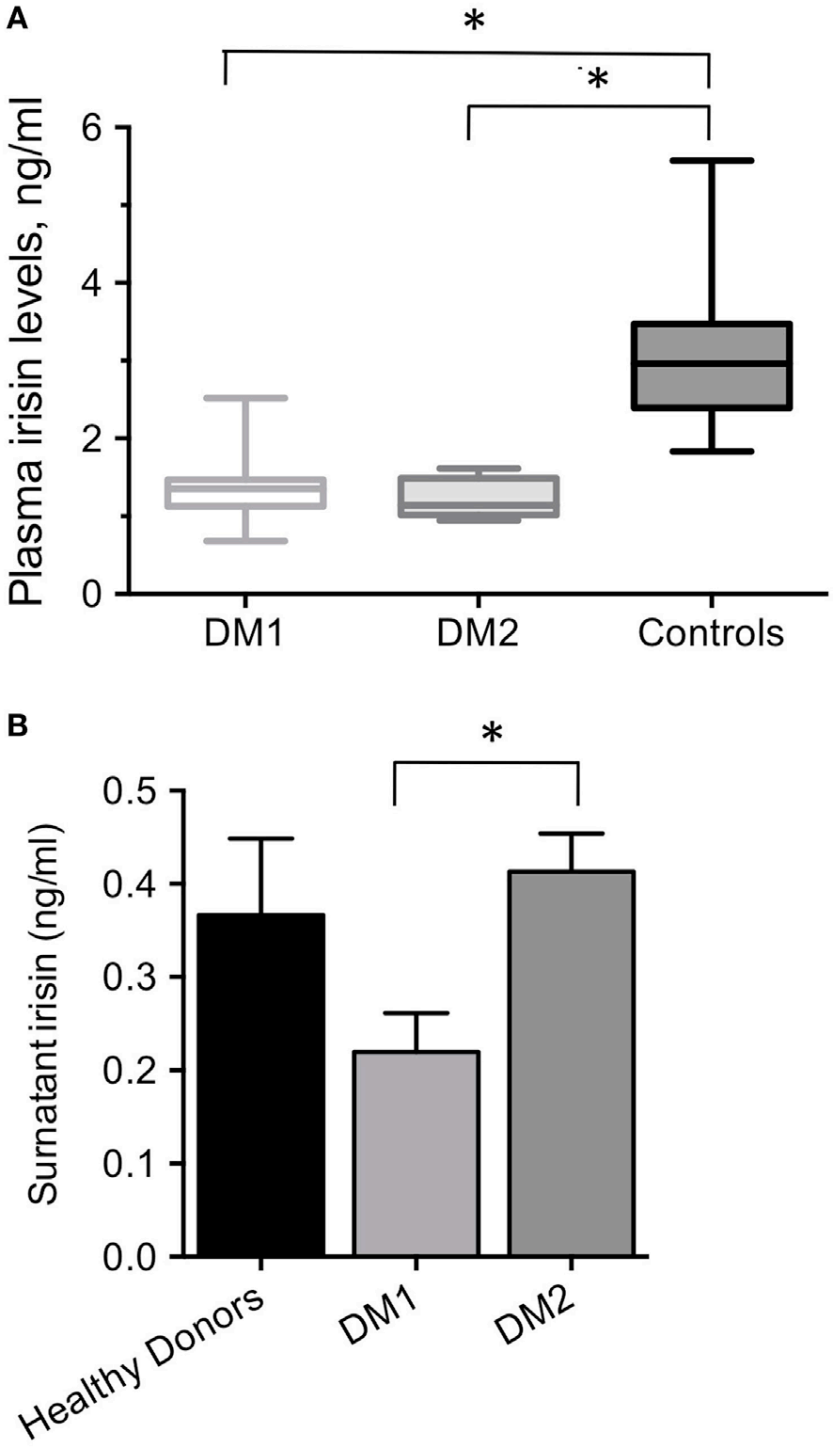
**(A)** Plasma irisin levels in DM1, DM2 male patients and age-matched, physically active, male healthy controls. *P < 0.0001 by ANOVA. **(B)** Surnatant irisin levels from 5 days-differentiated myotubes derived from DM1, DM2 patients and healthy controls; *P = 0.03.

At rest, plasma irisin levels detected in male healthy physically active subjects ranged from 1.8 to 5.6 ng/mL [3.0 (2.4–3.5) ng/mL, median, range interquartile]. Plasma irisin levels in DM1 patients ranged 0.68 to 2.52 ng/mL; median plasma irisin level in DM1 patients was 1.4 (1.1–1.5) ng/mL, and it was significantly lower than the median level detected in healthy controls (*P* < 0.0001) (Figure 1A). Plasma irisin levels detected in patients with DM1 did not differ significantly from levels detected in patients with DM2 [1.1 (1.0–1.5) ng/ml], which were definitely lower than levels detected in controls (Figure 1A).

### Irisin Release from Myotubes of DM1 and DM2 Male Patients

All starting cell populations used in this study had a myogenic purity higher than 80% (data not shown). All cultured myotube preparations from DM1, DM2 patients and control donors showed a fusion index of about 60% (data not shown). The DM2 myotubes were from patients with diet-treated diabetes, while both DM1 and control donors myotubes were from normoglycemic patients. Cultured myotubes derived from DM patients’ biopsies released detectable amounts of irisin in the medium (Figure 1B). Irisin released from DM1 myotubes was significantly lower than that released from the DM2 myotubes (0.22 ± 0.07 vs 0.41 ± 0.07 ng/ml; *P* = 0.03), though they both did not significantly differ from that released from healthy controls myotubes (0.33 ± 0.18 ng/ml; Figure 1B).

### Correlations between Plasma Irisin Levels and Metabolic Parameters in DM1 Patients

There was any significant correlation between plasma irisin levels and the age of patients at time of clinical evaluation or with the entity of triplet expansions. Any correlation could be detected between plasma irisin and parameters of muscle strength and performance, namely MRC and RMI (Table 1). Both total and segmental lean masses did not show any significant correlation with plasma irisin levels. Cardiac muscle mass has also been evaluated in DM patients by measurement of left ventricular mass by routine echocardiography (Table S1 in Supplementary Material): plasma irisin levels did not show any significant correlation with the left ventricular mass. Moreover, all the parameters describing fat mass (Table 1) were not correlated with irisin levels in DM1 patients, while a negative correlation was detected between plasma irisin levels and VO_2_ consumption (*r* = −0.429, *P* = 0.029).

**Table 1:** Clinical, anthropometric, hormone, and metabolic parameters in DM1, DM2, patients, and healthy controls.

Parameters	DM1 (*n* = 28)	DM2 (*n* = 10)	*P*-value*	Controls (*n* = 23)
Age	44.7 ± 11.5	56.7 ± 9.3	**0.005**	45.6 ± 14.5
BMI (kg/m^2^)	25.1 ± 4.1	26.8 ± 4.4	0.267	25.6 ± 3.06
Normal weight, % 18 < BMI ≤ 25	57	60	1.000	62
Overweight, % 25 < BMI ≤ 30	36	20	0.453	23
Obesity, % 30 < BMI ≤ 35	7	20	0.279	15

MRC scale	128.8 ± 14.9	145.4 ± 4.6	**0.002**	
MIRS scale	12.4 ± 3.4	14.5 ± 1.0	0.063	

Waist, cm	95.8 ± 10.2	97.2 ± 13.9	0.744	
Hip, cm	98.5 ± 8.4	98.1 ± 11.5	0.912	
Waist/hip	0.97 ± 0.05	0.99 ± 0.05	0.355	
VO2, mL/kg/min	2.5 ± 0.5	2.6 ± 0.3	0.754	
REE	1,351.7 ± 224.4	1,358.2 ± 280.7	0.942	
EF, systolic, mm	5.99 ± 2.90	6.00 ± 1.15	0.994	
Hepatic steatosis, %	57.1	70.0	0.269	

Glucose, mg/dL	79.6 ± 9.9	91.8 ± 24.3	0.033	
Insulin, microU/mL	10.7 ± 15.4	15.1 ± 11.7	0.422	
HOMA-IR	2.09 ± 2.78	3.73 ± 3.33	0.136	
Insulin resistance, %	14.3	60.0	**0.010**	
Diabetes mellitus, %	3.5	40.0	**0.012**	
HbA1c, %	5.29 ± 0.42	6.05 ± 1.38	**0.014**	
Total-C, mg/dL	203.6 ± 39.8	218.7 ± 41.3	0.314	
HDL-C, mg/dL	49.2 ± 11.6	52.6 ± 13.8	0.461	
LDL-C, mg/dL	125.9 ± 34.1	137.6 ± 34.7	0.363	
TG, mg/dL	145.9 ± 67.2	142.5 ± 94.7	0.902	
Dyslipidemia, %	53.6	80.0	0.259	

Testosterone, ng/dL	400.3 ± 147.6	344.2 ± 156.5	0.317	
Free-T, ng/dL	7.33 ± 3.31	5.78 ± 1.79	0.168	
17β-estradiol, pg/mL	46.4 ± 12.4	52.8 ± 11.5	0.180	
SHBG, nmol/L	42.6 ± 17.0	42.4 ± 22.4	0.976	
LH, mUI/L	8.5 ± 5.0	12.3 ± 5.8	0.057	
FSH, mUI/L	17.6 ± 13.1	31.9	**0.011**	
AMH, ng/mL	3.09 ± 3.42	0.64 ± 0.38	0.055	
Inhibin B, pg/mL	90.67 ± 107.64	37.51 ± 24.54	0.181	
Hypogonadism, %	25.0	50.0	0.235	

*BMI, body mass index; MRC, modified 5-point Medical Research Council scale; MIRS, muscular impairment rating scale; REE, resting energy expenditure; EF, epicardial fat; C, cholesterol; TG, triglycerides; free-T, free testosterone; SHBG, sex-hormone binding globuline; LH, luteinizing hormone; FSH, follicle-stimulating hormone; AMH, anti-Müllerian hormone*.

*Comparison between DM1 and DM2 patients.

*Bold means “statistically significant differences”*.

**Figure 2:**
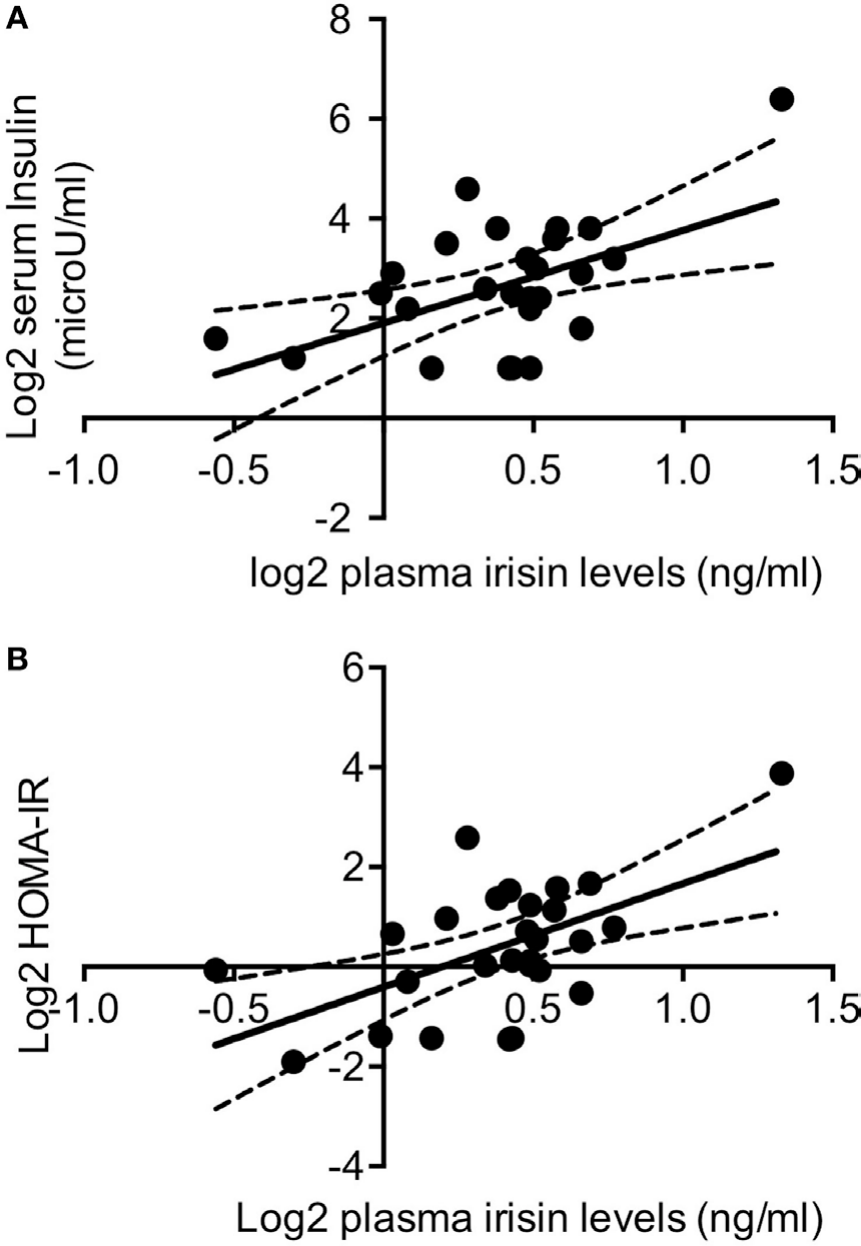
Log2-transformed irisin levels positively correlated with log2 serum insulin levels **(A)** and with log2 HOMA-IR values in non-diabetic DM1 patients (*n* = 27) **(B)**; continuous lines represent media values, dashed lines represent the 95% confidence intervals.

In normoglycemic DM1 patients (*n* = 27), plasma irisin levels positively correlated with insulin (*r* = 0.457; *P* = 0.017; Figure 2A) and HOMA-IR (*r* = 0.428; *P* = 0.026; Figure 2B). Any significant correlation was detected between plasma irisin and serum glucose as well as with lipid profile, namely serum total cholesterol, HDL cholesterol, LDL cholesterol, and triglycerides levels (Table 1). Similarly, there was any correlation with both Sertoli and Leydig cell hormones, namely, serum total and free testosterone, serum inhibin B and AMH levels (Table 1).

Plasma irisin levels were positively correlated with serum 25OHD in DM1 patients (*r* = 0.451; *P* = 0.018); indeed, after adjustment for the fat mass, the correlation was no longer significant suggesting that it is mediated by the known correlation with the fat mass (23). Finally, in normoglycemic DM1 patients plasma irisin positively correlated with pelvis BMD (*r* = 0.622, *P* = 0.041) and mean legs BMD (*r* = 0.569, *P* = 0.045) (Table 2).

**Table 2:** Bone metabolism parameters and regional bone mineral densities evaluated by DEXA in DM1 and DM2 patients.

	DM1 (*n* = 15)	DM2 (*n* = 10)	*P*-value
**Bone metabolism parameters**			
Alb-corrected calcium, mg/dL	9.3 ± 0.4	9.3 ± 0.5	0.887
Phosphate, mg/dL	2.96 ± 0.58	3.29 ± 0.43	0.113
ALP, U/L	85.8 ± 35.2	64.8 ± 11.7	0.075
PTH, pg/mL	51.9 ± 41.5	57.2 ± 24.0	0.704
25OHD, ng/mL	16.4 ± 13.2	16.7 ± 10.4	0.960
**Bone mineral density (BMD)**			
BMD mean arms, g/cm^2^	0.84 ± 0.08	0.76 ± 0.06	**0.037**
BMD mean ribs, g/cm^2^	0.77 ± 0.27	0.67 ± 0.09	0.390
BMD T spine, g/cm^2^	0.90 ± 0.28	0.91 ± 0.12	0.904
BMD L spine, g/cm^2^	1.14 ± 0.13	1.08 ± 0.14	0.391
BMD pelvis, g/cm^2^	1.27 ± 0.18	1.05 ± 0.08	**0.008**
BMD mean legs, g/cm^2^	1.28 ± 0.14	1.17 ± 0.08	0.069
BMD subtotal, g/cm^2^	1.10 ± 0.11	0.97 ± 0.06	**0.014**
BMD head, g/cm^2^	2.12 ± 0.49	1.88 ± 0.24	0.243
BMD total, g/cm^2^	1.21 ± 0.13	1.09 ± 0.06	**0.039**

*Alb-corrected calcium, albumin corrected calcium; ALP, alkaline phosphatase activity; PTH, parathormone; 25OHD, 25 hydroxyvitamin D; T spine, thoracic spine; L spine, lumbar spine*.

*Bold means “statistically significant differences”*.

### Correlations between Plasma Irisin Levels and Metabolic Parameters in DM2 Patients

**Figure 3:**
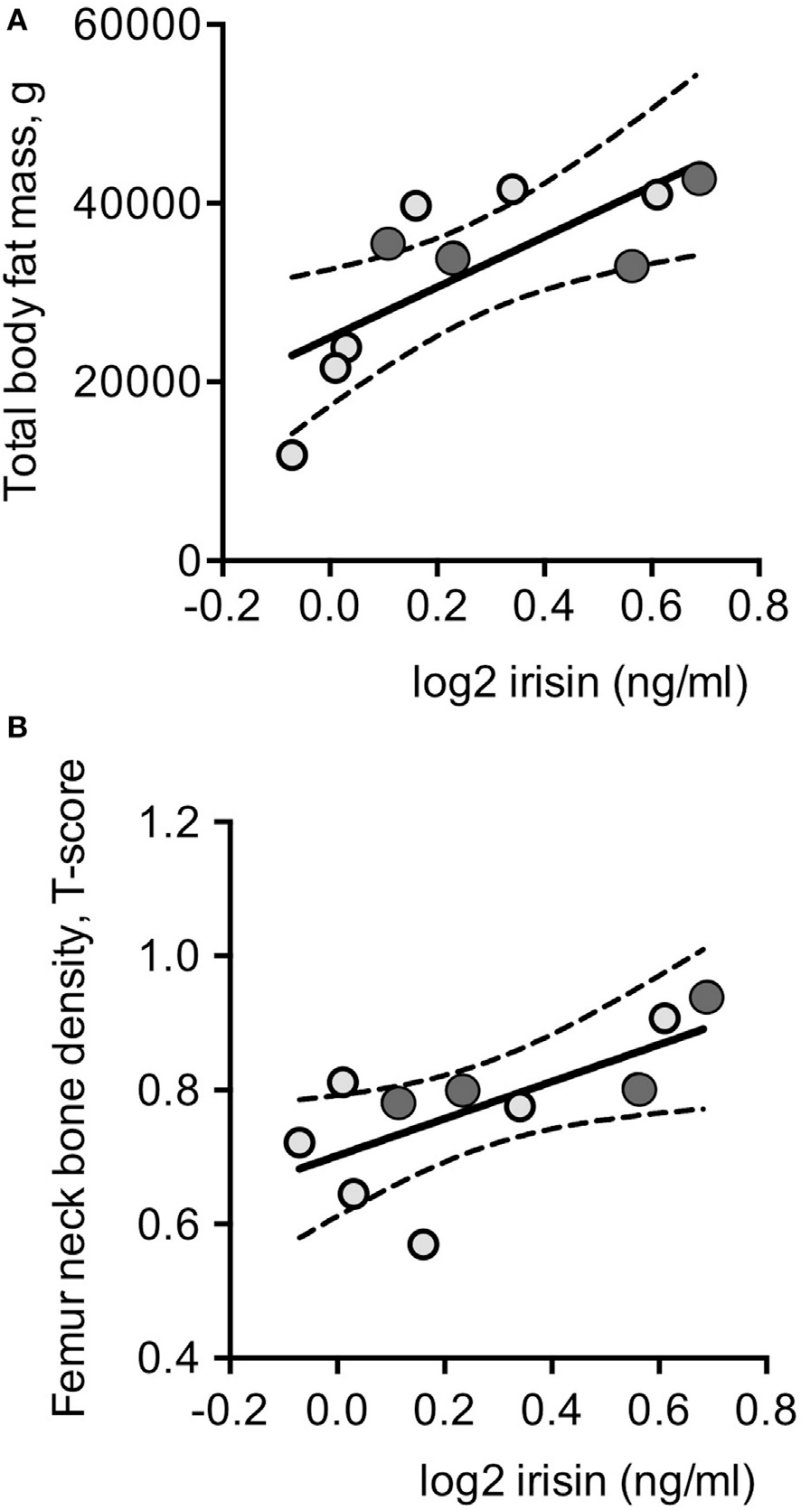
Log2-transformed irisin levels positively correlated with total body fat **(A)** and with femoral neck T-score values in the whole series of DM2 patients (*n* = 10) **(B)**. Darker circles indicate DM2 patients with diabetes mellitus; continuous lines represent media values, dashed lines represent the 95% confidence intervals.

Plasma irisin levels in DM2 patients did not correlate with age, and parameters of muscle strength and performance, MRC and RMI, as observed in DM1 patients (Table 1). Though total and segmental lean masses, as well as cardiac left ventricular mass, did not show any significant correlation with plasma irisin levels in DM2 patients (Table 1; Table S1 in Supplementary Material), total fat mass (Figure 3A), and fat mass at arms and legs levels positively correlated with plasma irisin levels (Table 3).

**Table 3:** Significant correlations between plasma irisin levels and regional and total fat measured by DEXA in non-diabetic DM2 patients.

Parameters	*r*	*P*-value
Fat left arm	0.934	0.020
Fat right arm	0.976	0.005
Fat left leg	0.981	0.003
Fat right leg	0.991	0.001
Fat total body	0.830	0.041

At variance with DM1 patients, any significant correlation could be detected with parameters of insulin resistance and glucose metabolism as well as with lipid profile, gonadal function, and vitamin D status in normoglycemic DM2 patients (Table 1). Plasma irisin positively correlated with both *T*-score (*r* = 0.679, *P* = 0.031; Figure 3B and Table 2) and *Z*-score (*r* = 0.719, *P* = 0.019) at femur neck level in DM2 patients. Indeed, considering normoglycemic DM2 patients (*n* = 6) in order to exclude the effect of diabetes, the correlation could not be further detected, likely due to the small sample size.

## Discussion

Myokines modulate processes related to energy metabolism and muscle regenerative capacity in active muscle; moreover, they modulate plasticity of other organs and tissues as a part of the adaptive response to acute and/or regular exercise. The discovery of irisin raised great interest in the scientific community for its role as mediator of the cross talk between muscle and fat (24) and, more recently, between muscle and bone (25, 26). Nonetheless, some Authors claimed that the poor antibodies specificity against irisin give artifacts in measuring human irisin; indeed, by using an unbiased assay, human irisin has been demonstrated and quantitated in human plasma samples (5). The ELISA assays used in the present study detected irisin concentrations in the plasma samples from the male healthy controls similar to those detected by tandem mass spectrometry in sedentary males (5).

Though the role of irisin in clinical diseases is almost unknown, its functional characteristics suggest it may be involved in muscle dystrophies. Patients with DM1 and DM2 have muscle wasting and sarcopenia, involving proximal or distal segments of the extremities, respectively. In the present study, we first investigated circulating levels of the myokine irisin in male DM patients in resting conditions and reported that plasma irisin was definitely decreased in both DM1 and DM2 patients compared with age-matched male healthy controls. We investigated whether this reduction was related to muscle mass reduction or to an impaired muscle endocrine function in DM patients. The amount of irisin released from cultured myotubes derived from DM1 and DM2 patients was unexpectedly similar to that detected in the medium of the myotubes derived from healthy donors, suggesting that the decreased plasma irisin levels likely reflect the skeletal muscle mass reduction rather than an impaired endocrine function of the muscle fibers. Indeed, irisin release from DM2 myotubes, whose patients had overt diabetes mellitus, was significantly higher than the amount released from DM1 non-diabetic myotubes. It is worth noting that myotubes from patients with type 2 diabetes expressed and secreted the highest levels of *FNDC5* mRNA and irisin, respectively (27), resembling our finding in DM2 patients.

Although DM1 and DM2 have similar symptoms, they present dissimilarities making them clearly distinct diseases (28). Distal muscle weakness, facial weakness, and wasting are the core features in adult-onset DM1, while DM2 patients experience varying grip myotonia, thigh muscle stiffness, and muscle pain, as well as proximal muscle weakness (28). Besides myotonia and progressive muscle weakness, DM1 and DM2 are multisystemic syndromes and multiple metabolic functions are impaired. The analysis of the correlations between plasma irisin levels and anthropometric and metabolic parameters showed a distinct pattern in DM1 and DM2 patients, respectively, in DM1 patients, plasma irisin levels correlated negatively with oxygen consumption and positively with insulin resistance, while in DM2 patients plasma irisin levels positively correlated with fat mass at arms and legs levels.

To discuss in detail our finding, it has to be considered that the main sources of circulating irisin are skeletal muscle, cardiac muscle (29) and white adipose tissue. First, despite in a cohort of sedentary middle-aged men, circulating irisin was positively associated with muscle mass and muscle strength (30), both DM1 and DM2 patients did not present any significant correlation with muscle strength, muscle mass, or measures of disease severity. Second, knockdown of irisin in zebrafish decreased heart rate, diastolic volume, and cardiac output (29). DM1 and DM2 patients suffer from cardiac damage including abnormalities of the conduction system (31) and myocardial fibrosis (32, 33); though left ventricular mass was reduced in more than 60% of DM patients, in agreement with previous reports (33), plasma irisin did not correlate with left ventricular mass in both DM1 and DM2 patients. Third, adipose tissue expresses the *FNDC5* gene and secretes irisin, but less than skeletal muscle (34) and circulating irisin levels are positively associated with fat mass (20, 35–38), while negatively correlate with visceral adiposity in men (30). In our cohort, both DM1 and DM2 patients were characterized by a fat redistribution with increasing visceral fat mass, namely abdominal, liver, epicardial fat, while subcutaneous fat was reduced (16, 39). Similar to what reported in healthy subjects, DM2 patients were characterized by a positive correlation between plasma irisin levels and the arms and legs fat measured by DEXA, while in DM1 patients, no correlation could be detected with the total body and regional fat mass as well as with visceral adiposity parameters, such as waist circumference, liver steatosis, and epicardial fat thickness.

Secreted irisin targets different tissues, mainly white adipose tissue and bone. In white adipose tissue, irisin induces browning, increases thermogenesis and energy expenditure. Oxygen consumption is reduced in DM1 patients (16), and here we reported that it negatively correlated with plasma irisin levels, suggesting that irisin does not exert the expected thermogenic effect on adipose tissue in DM1 patients. Bone is a recently described target of irisin, which plays a role in the control of bone mass. Irisin exerts its effect mainly on osteoblast lineage by enhancing differentiation and activity of bone-forming cells (40). In humans, an inverse correlation between irisin levels and vertebral fragility fractures was described in overweight subjects, though BMD or lean mass were not correlated with irisin levels (41, 42). In both DM1 and DM2 patients, we observed positive correlations between plasma irisin and bone densities at pelvis and legs and at the femoral neck levels, respectively. Though the effect of diabetes could not be excluded in DM2 patients, this finding support the irisin-mediated cross talk between muscle and bone.

Finally, male DM1 and DM2 patients are metabolically unhealthy; in particular, they are hypogonadic, insulin resistant, and dyslipidemic. Hypogonadism frequently occurs in DM1 and DM2 patients (16), nonetheless plasma irisin levels were not correlated with parameters of gonadal hormone function. The finding is in agreement with experimental evidence reporting that orchiectomy in rats does not affect serum irisin levels and testosterone treatment in orchiectomized rats does not affect muscle *Fdnc5* expression as well as serum irisin levels (43). Insulin resistance occurs in 25.0% of DM1, while overt diabetes was diagnosed in 3.5% of DM1. Recently, in sedentary subjects and in non-diabetic adults, plasma irisin was found to positively correlate with insulin and HOMA-IR (44, 45). Similarly, we observed a positive correlation between plasma irisin and insulin and HOMA-IR values in non-diabetic DM1 patients, while insulin resistance, which could be detected in 60.0% of non-diabetic DM2 patients, did not show any significant correlation with plasma irisin levels.

In conclusion, DM1 and DM2 male patients were characterized by reduced plasma irisin, which likely reflects muscle wasting as DM1 and DM2 muscle fibers conserve the ability to secrete irisin. Admittedly, though the series of DM1 and DM2 patients are well characterized about the endocrine and metabolic phenotype, the small sample size prevents to define the role of insulin resistance and hyperglycemia in regulating plasma irisin levels. However, though mainly based upon correlations, our results suggest that muscle endocrine function may be more impaired in DM1 patients than in DM2 patients.

Based on recently reported results in mice (26), we speculate that replacement with recombinant irisin in young DM patients might improve their functional and metabolic profile; these prospective view is also sustained by *in vitro* data showing that irisin stimulates myogenesis, as suggested by increased myocyte cell proliferation, higher myogenin/*MYOG* mRNA levels together with lower transcripts of myostatin/*MSTN* and dystrophin/*DMD*, and the muscle atrophy-related factors *MuRF1* and *MAFbx* (5, 46). Considering that during differentiation DM1 and DM2 myotubes do not increase myogenin (47), treatment with recombinant irisin may be of interest in DM1 and DM2 management.

## Ethics Statement

All the participants gave their written informed consent and the local ethical committee approved the study protocol. The study complied with the Declaration of Helsinki.

## Author Contributions

EP, SB, CA, and VS enrolled and collected clinical data, serum samples, and muscle biopsies from patients and controls; RV performed genetic diagnosis; RC realized myotubes characterization and cultures and collected supernatant samples; ED performed ELISA assays; MC, GM, and SC supervised and checked all the experiments; SC and VS conceived the protocol, ensured the accuracy and the integrity of the work, and wrote the manuscript. All the Authors critically reviewed and approved the manuscript draft.

## Conflict of Interest Statement

The research was conducted in the absence of any commercial or financial relationships that could be construed as a potential conflict of interest.
