# Supplementary material for: Circulating Irisin Is Reduced in Male Patients with Type 1 and Type 2 Myotonic Dystrophies
Source: Front Endocrinol (Lausanne). 2017 Nov 14;8:320. doi: 10.3389/fendo.2017.00320 (PMC5694592; doi:10.3389/fendo.2017.00320)
Supplement: Supplementary file 1 [file table_1.pdf]

Supplemental Table 1. Routine echocardiographic parameters in DM patients.

	DM1	DM2	P
LVIDd, cm	4.8±0.4	4.7±0.4	0.850
LVIDs, cm	3.2±0.5	3.2±0.5	0.816
FS,%	32.3±7.4	33.3±6.7	0.745
RWT, mm	0.40±0.08	0.44±0.08	0.205
LVMI index, g/cm^2	89.0±18.2	103.9±30.0	0.101
LVEF, %	60.2±7.3	57.3±8.2	0.350
IVSd, mm	0.98±0.16	1.10±0.13	0.073
IVSs, mm	1.29±0.23	1.51±0.20	**0.018**
E/A ratio	1.34±0.40	1.04±0.26	0.08

LVIDd: left ventricular internal diameter in diastole; LVIDs: left ventricular internal diameter in systole; FS: fractional shortening; RWT: relative wall thickness; LVMI index: left ventricular mass index; LVEF: left ventricular ejection fraction; IVSd: diastolic interventricular septal thickness; IVSs: systolic interventricular septal thickness; E/A, early (E) to late (A) ventricular filling velocities.
